# Supplementary figures and images for: Drosophila ZDHHC8 palmitoylates scribble and Ras64B and controls growth and viability
Source: PLoS One. 2019 Feb 8;14(2):e0198149. doi: 10.1371/journal.pone.0198149 (PMC6368284; doi:10.1371/journal.pone.0198149)

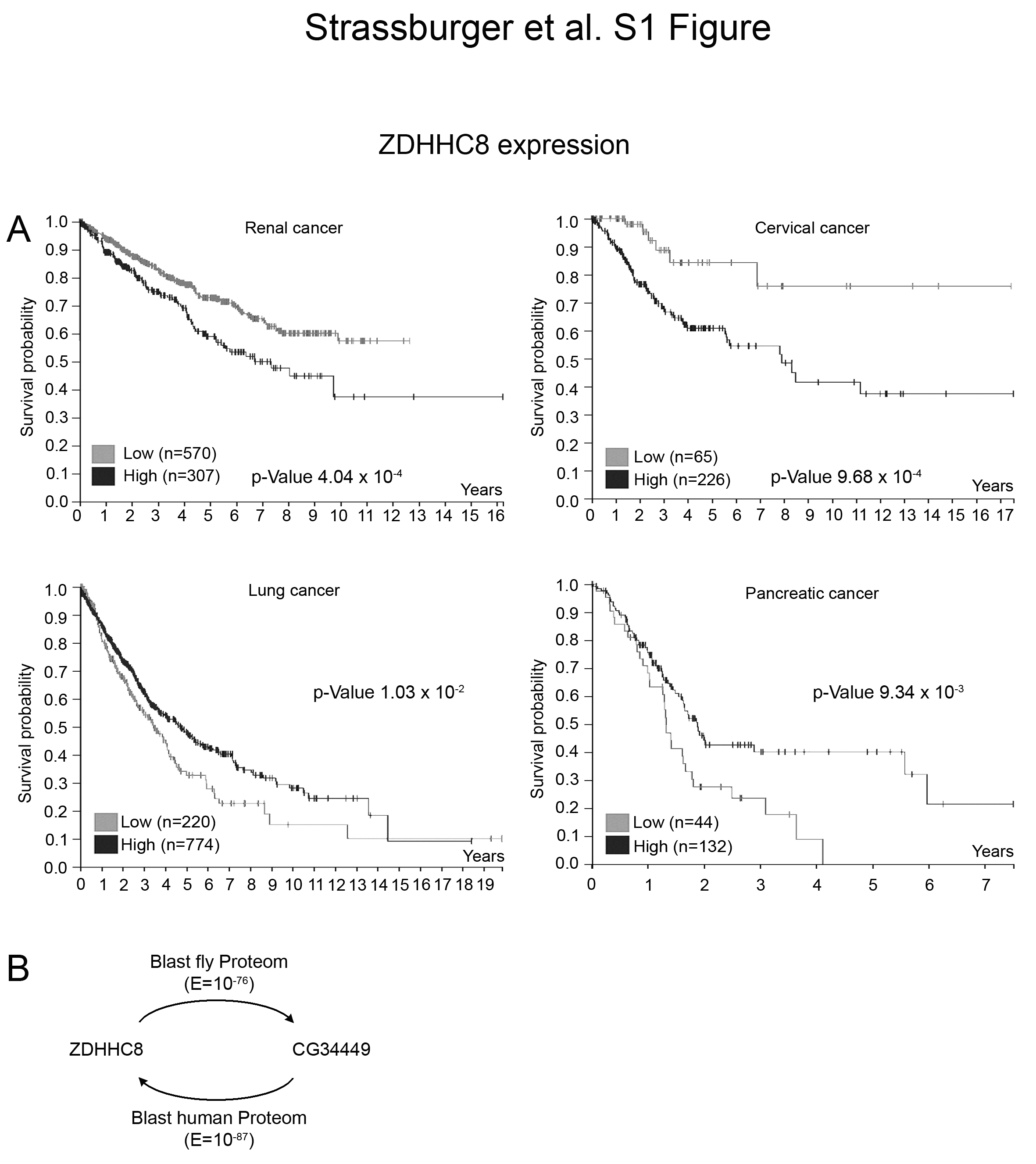

Supplement: S1 Fig — (A) ZDHHC8 expression levels correlate with increased or reduced cancer survival depending on cancer type. Kaplan-Meier plots (best separation, taken from The Human Protein Atlas using data from The Cancer Genome Atlas https://cancergenome.nih.gov) show a correlation between ZDHHC8 expression and patient survival for different cancer types. In renal (log-rank P value 4.04 x 10^−4^) and cervical cancer (log-rank P value 9.68 x 10^−4^) high ZDHHC8 expression correlates with decreased survival, whereas in lung (log-rank P value 1.03 x 10^−2^) and pancreatic cancer (log-rank P value 9.34 x 10^−3^) low ZDHHC8 expression correlates with decreased survival. (B) BLAST search using the Flybase BLAST server [37] of the Drosophila proteome using the protein sequence of human ZDHHC8 (NCBI Reference Sequence NP_037505.1) yields CG34449 as the top hit, with an E value of 10−76. Conversely, BLASTing the protein sequence of Drosophila CG34449 against the human proteome yields ZDHHC8 as the top hit with an E value of 10−87, identifying ZDHHC8 as the human orthologue of Drosophila CG34449. (TIF) [file pone.0198149.s001.tif]

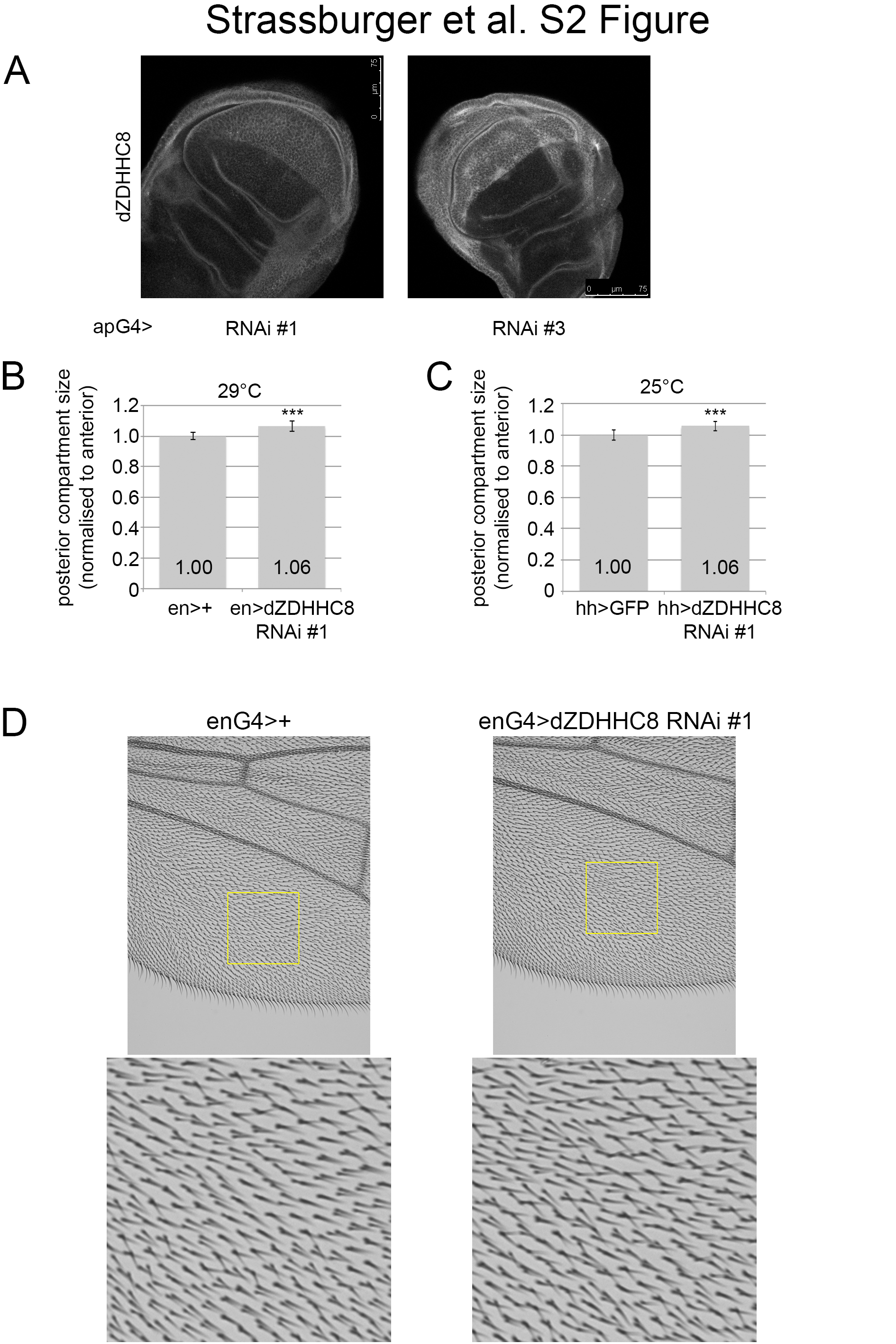

Supplement: S2 Fig — (A) Wing discs with dZDHHC8 knockdown in the dorsal compartment using apterous-GAL4 (apG4), stained anti dZDHHC8. (B) Expression of dZDHHC8 RNAi in the posterior compartment of the wing using engrailed-Gal4 (en>dZDHHC8 RNAi #1) at 29°C causes an increase in posterior compartment size normalized to anterior when compared to control wings (enG4>+). Error bars = stdev. n = 10. *** ttest = 1x10^-4^. (C) Expression of dZDHHC8 RNAi in the posterior compartment of the wing using hedgehog-Gal4 (hh>dZDHHC8 RNAi #1) causes an increase in posterior compartment size normalized to anterior when compared to control wings (enG4>+). Error bars = stdev. n = 10. *** ttest = 1x10^-3^. (D) Representative examples of wings used to quantify cell size in Main 1C. Quantified regions are marked with a yellow box, and shown in higher magnification underneath. (TIF) [file pone.0198149.s002.tif]

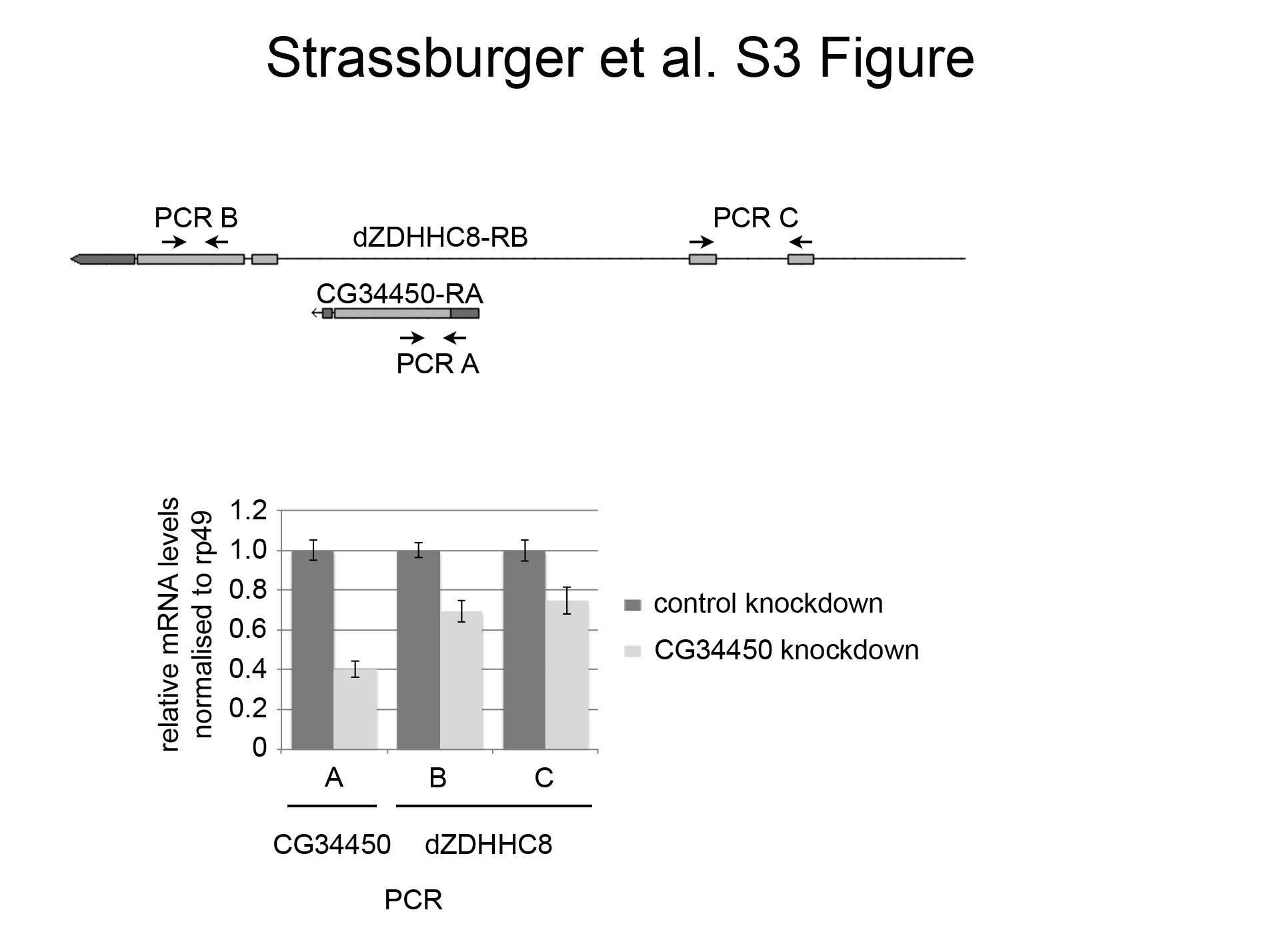

Supplement: S3 Fig — The expression of different dZDHHC8 exons (PCR B and C) was analysed by quantitative RT-PCR in control cells (dark grey bars) or cells with a CG34450 knockdown (light grey bars). When CG34450 is knocked down, transcript levels of dZDHHC8 also decrease suggesting dZDHHC8 and CG34450 are not separate genes. (TIF) [file pone.0198149.s003.tif]

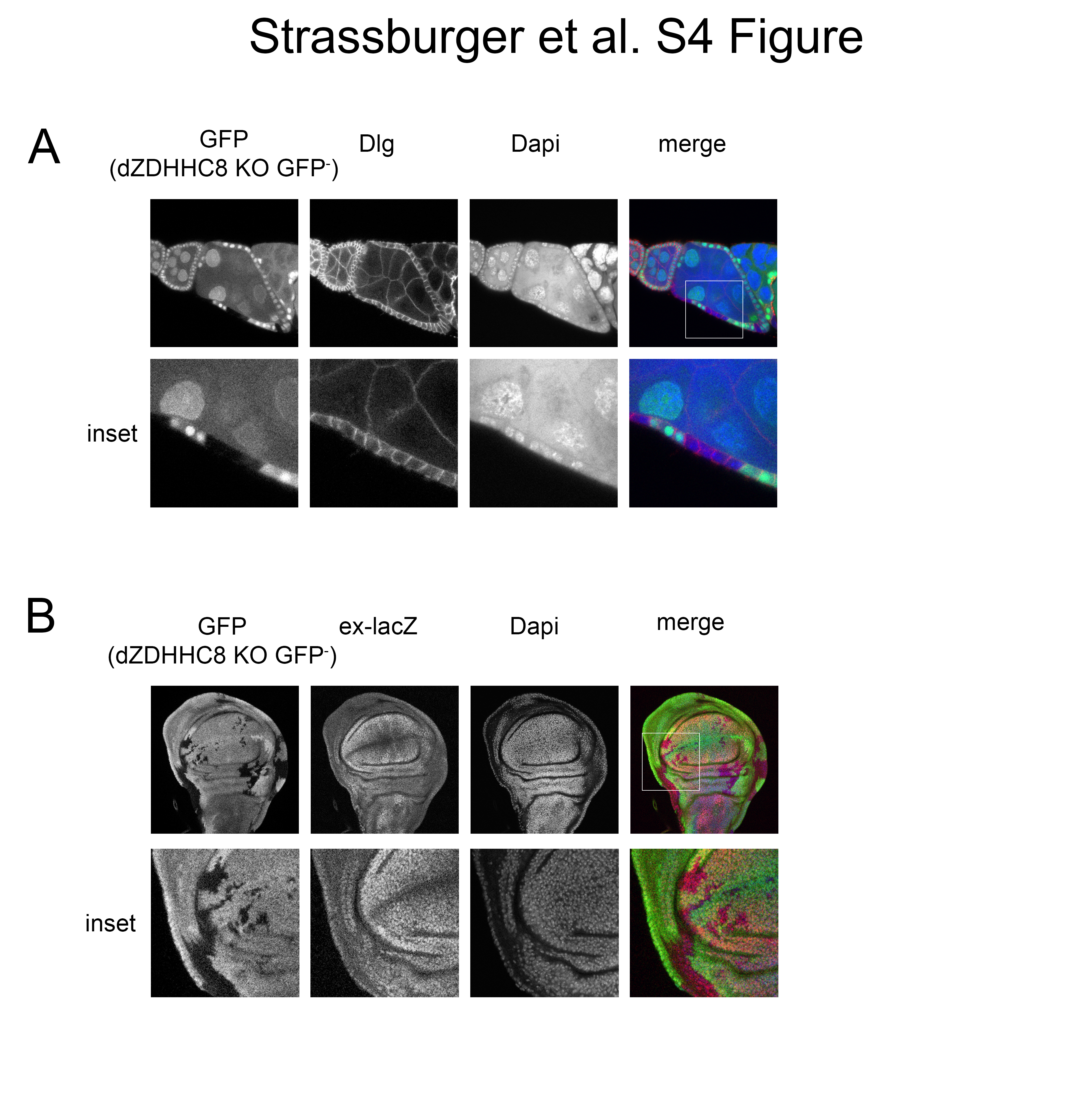

Supplement: S4 Fig — (A) dZDHHC8 mutant follicle clones (GFP negative) were stained with an antibody detecting endogenous Dlg (red). Dlg localization is not affected in dZDHHC8 mutant clones. (B) Three day old dZDHHC8 knockout clones (GFP negative) in the wing disc were stained for the yorkie reporter ex-lacZ (red). Yorkie activity is not affected in dZDHHC8 mutant clones. (TIF) [file pone.0198149.s004.tif]
